# Nutrient and Phytochemical Composition of Nine African Leafy Vegetables: A Comparative Study

**DOI:** 10.3390/foods14081304

**Published:** 2025-04-09

**Authors:** Robert Lugumira, Henry Tafiire, Flore Vancoillie, Geoffrey Ssepuuya, Ann Van Loey

**Affiliations:** 1 Laboratory of Food Technology, Department of Microbial and Molecular Systems (M2S), KU Leuven, Kasteelpark Arenberg 22, P.O. Box 2457, 3001 Leuven, Belgium; robert.lugumira@kuleuven.be (R.L.); henry.tafiire@kuleuven.be (H.T.); flore.vancoillie@kuleuven.be (F.V.); 2 Department of Food Science and Technology, Kyambogo University, Kyambogo, Kampala P.O. Box 1, Uganda; gksepuya@gmail.com

**Keywords:** leafy vegetables, minerals, vitamin C, carotenoids, polyphenols, phytochemicals

## Abstract

Micronutrient deficiencies and the increased incidences of non-communicable diseases are public health challenges for the sub-Saharan population. Leafy vegetables reportedly contain several minerals, vitamins and antioxidant compounds which could help reduce the above challenges. However, overall vegetable consumption is still low in Uganda, partly due to limited information about the nutritional quality and health-promoting properties of the available vegetable species. To provide scientific justification for increased growth/production and utilisation of specific leafy vegetable species as food, the nutrient and phytochemical compositions were determined in nine African leafy vegetables. The ‘true’ protein and dietary fibre contents were 13–33 and 29–43 g/100 g DW, respectively, whereas the starch content was below 6 g/100 g DW. As for the minerals, the Ca, Mg, and Fe contents were 630–3395, 324–1428, and 14–78 mg/100 g DW, respectively, whereas the Zn content was below 6 mg/100 g DW. High carotenoid contents were observed with lutein and β-carotene as the predominant carotenoids (25–60 and 12–29 mg/100 g DW, respectively). The vitamin C and total polyphenol contents were 24–253 mg/100 g DW and 17–43 mg GAE/g DW, respectively. The leafy vegetables studied are low-calorie and can be considered alternative protein sources. They are generally health-beneficial foods as they contain natural antioxidant compounds, vitamin C, carotenoids and polyphenols. African nightshade, *Amaranthus* sp., cowpea leaves, and spider plant are potential sources of provitamin A (β-carotene) and minerals (Ca and Fe); hence, they can help reduce incidences of related deficiencies.

## 1. Introduction

Africa is endowed with several plant species whose leaves have been utilised as food for centuries [1]. These are referred to as African leafy vegetables (ALVs), which include leaves of cultivated plant species such as beans, cowpeas, yams, and pumpkins, and non-cultivated (wild and weedy) plant species [2]. Generally, these leafy vegetables are mainly consumed as accompaniments, side dishes or sauces of the main course meals prepared from staple foods such as cooking bananas, cassava, and sweet potatoes, among others. During staple food crop shortages, they play a key role in communities where they are used as accompaniments because they become major components of the meals. In the past, most of these vegetables would be collected from the wild; however, increased urbanisation (disappearance of the wild), the formalisation of agriculture, and awareness of their pro-health properties and medicinal values have led to farm-yard (home) cultivation and large-scale production for commercial gains [1,3]. Currently, they are mainly obtained from farms (own production) and/or fresh produce markets (urban setting), and to a smaller extent, the wild. In the Ugandan context, Sseremba et al. [3] highlight seven major plant families of importance as vegetables, five of which are families of African leafy vegetables, including *Solanaceae*, *Amaranthaceae*, *Malvaceae*, *Fabaceae*, and *Cucurbitaceae*.

Although ALVs are poor carbohydrate and fat sources, they are reported to contain relatively high amounts of protein (up to 33% DW) [4], dietary fibre [5], minerals (Ca, Mg, Fe, and Zn, among others) and vitamins (A, E, and C) [6,7,8,9]. They also contain compounds with antioxidant and anti-inflammatory properties such as carotenoids, ascorbic acid, and polyphenols (flavonoids and phenolic acids), which can scavenge free radicals [10]. Studies suggest an association between these phytochemicals and a low risk of degenerative diseases such as cardiovascular diseases [10,11,12]. A meta-analysis by Kiyimba et al. [11] on the effectiveness of polyphenols in optimising cardiometabolic health showed that food polyphenols can reduce cardiometabolic risks and help optimise health. A reduced risk of non-communicable diseases (NCDs) has been associated with antioxidant compounds, which are predominantly plant-based. Nonetheless, the concentration of these nutrients and phytochemicals varies in different vegetables due to differences in maturity, plant cultivar, and agro-climatic conditions, among others [12,13,14].

In sub-Saharan Africa, specifically in East Africa, there is low vegetable consumption and high dependence on energy-dense staples such as cooking bananas, cassava, potatoes, and foods from refined cereals, which are deficient in essential micronutrients [15,16]. This could explain the high prevalence of micronutrient deficiencies in the region, especially for iron, calcium, and vitamin A [17]. Additionally, there is a reported growing risk of NCDs in developing countries, particularly in the urban areas of Uganda, where the population heavily relies on energy-dense fast foods made from refined raw materials [18].

Since food security cannot exist without nutrition security, solutions are needed to boost vegetable production and make the staple food-loaded diets in Uganda more nutritious and healthier. ALVs with a short maturation period, limited farming management costs, and contain substantial amounts of nutritionally vital components [7] could be a viable option, but they are still underutilised in communities where they would be very beneficial. On a positive note, if a population gains more knowledge about the health benefits of vegetables, the consumption rate can gradually increase [18]. Therefore, this study looks at the nutritional quality (macro and micronutrients) and health-related compounds in the most consumed ALVs in Uganda. It provides a scientific justification for the increased growth/production and utilisation of specific leafy vegetable species as food. This provides a more sustainable solution to the protein and micro-nutrient-based burden of malnutrition and increases the intake of natural antioxidant compounds for their health-promoting properties.

## 2. Materials and Methods

### 2.1. Acquisition of Vegetable Samples

Uganda has several leafy vegetables that are traditionally preferred for consumption in different regions. To select vegetables to be considered for this study, a survey was conducted to collect information about the vegetables consumed in the four regions of Uganda (the Eastern, Western, Northern and Central regions). Using the snow sampling technique, about 100 key informants were interviewed in each region, chosen based on their cultural background and knowledge about traditional vegetables consumed in their area. The collected information included the vegetables consumed, the consumption frequency, the processing methods used, and the methods used to improve shelf life. Based on this survey and a study by Sseremba et al. [3], nine leafy vegetables that are commonly consumed were selected. These were African nightshade (*Solanum aethiopicum*), nightshade (*Solanum nigrum*), green amaranthus (*Amaranthus dubius*), red amaranthus (*Amaranthus cruentus*), collard leaves (*Brassica oleraceae* var *acephala*), cowpea leaves (*Vigna unguiculata*), Malakwang (*Hibiscus sabdariffa*), pumpkin leaves (*Curcubita maxima*), and spider plant (*Cleome gynandra*). These leafy vegetables (5 kg each) were collected from the markets in Kampala, Uganda (January 2023), since the population mainly obtains its vegetables from markets, not through self-production.

### 2.2. Sample Preparation

On reception, the vegetables were cleaned with potable running water to ensure all the visible dirt (soil) was washed off, and the excess water was removed using blotting paper. The leaves were separated from the shoots by finger plucking and frozen to −40 °C. The frozen leaves were freeze-dried (YK-118 Vacuum Freeze Dryer, Taiwan), and the lyophilised vegetables were packaged in tight zipper bags, shipped to Belgium, and stored in the dark at −40 °C while awaiting analysis. Noteworthy is that before any analysis, the freeze-dried leaves were mechanically ground into a fine powder with a ball mill, MM400 (Retsch GmbH, Haan, Germany), for 30 s at a frequency of 30 s^−1^.

### 2.3. Macronutrient Composition Analysis

Immediately after sample reception, the moisture content of the fresh samples was quantified as the weight loss upon convection air drying at 105 °C for 16 h [19] in an oven (UN750 Memmert GmBH, Schwabach, Germany). The total nitrogen content was analysed using automated Dumas combustion analysis (CE instrument, Thermo-Fischer Scientific, Waltham, MA, USA) (AOAC, method 990.03). Since no specific conversion factor is available for leafy vegetables, 6.25 [20] was used to calculate the crude protein contents. After trichloroacetic acid precipitation, the true protein content of the vegetables was determined as described by Vanleenhove et al. [21]. Briefly, to 1 g of the ball milled sample, 15 mL of 20% trichloroacetic acid was added and vortexed for 1 min. The samples were put in an ice bath for 1 h to precipitate soluble proteins and then centrifuged at 3020× *g* for 10 min (Avanti JXN-26, Beckman Coulter Inc, Indianapolis, IN, USA). The pellet containing the protein was freeze-dried using Alpha 2-4 LSC plus (Christ, Osterode, Germany) and the true protein content was determined using the Dumas method. The total dietary fibre was quantified using the Total Dietary Fibre test kit (Megazyme Inc. Bray, Ireland) (AOAC, method 985.29), and the total starch kit (AA/AMG, Megazyme Inc. Bray, Ireland) (AOAC, method 996.11) was used to determine the total starch content. The total ash content was quantified by incinerating 1 g of the samples at 550 °C for 23 h, and the residue in the crucible was weighed as the total ash (AOAC method 923.03).

### 2.4. Micronutrient Composition Analysis

#### 2.4.1. Mineral Analysis

The mineral analysis for the vegetable samples was performed using an inductively coupled plasma-optical emission spectrometer (ICP-OES) (iCAP 7400 Duo spectrometer, Thermo Fisher Scientific, Newington, CT, USA), as elaborated by Gwala et al. [22]. Briefly, 20 mg of each freeze-dried sample was completely incinerated at 550 °C in a muffle furnace for 20 h. The residue ashes were then dissolved in 10 mL of acidified (1% nitric acid) Milli-Q water (18.2 M·.cm). The solutions were filtered through a 0.45 µm membrane and the dissolved minerals were quantified using ICP-OES.

#### 2.4.2. Vitamin C Analysis

Vitamin C extraction and quantification were performed following the method elaborated by Verbeyst et al. [23]. A sample of 200 mg was weighed into a centrifuge tube followed by 10 mL of extraction buffer (1% m-phosphoric acid and 0.5% oxalic acid adjusted to pH 2). The mixture was then vortexed for one minute and centrifuged at 19,900× *g* and 4 °C for 30 min in a Sigma 4-16KS centrifuge (Osterode, Germany). A portion of the supernatant (5 mL) was taken, and its pH adjusted to 3.5 with 1 N sodium hydroxide. From this, an aliquot of 1 mL was mixed with 2 mL of reducing agent in phosphate buffer [2.5 mM Tris-2-carboxy-ethyl Phosphine (TCEP)] to convert the dehydroascorbic acid in the extract to ascorbic acid. The resultant solution was centrifuged for 15 min at 19,900× *g*, 4 °C, and then filtered through a 0.45 µm membrane into amber HPLC vials to determine the total vitamin C content.

The determination of the vitamin C content was performed using a Prevail C18 column (250 × 4.6 mm, 5 μm particle diameter, Theale, England) with a guard column and an HPLC system coupled to a diode array detector (1200 Series, Agilent Technologies, Diegem, Belgium) set at 245 nm. An isocratic elution buffer (1 mM ethylene diamine tetraacetate disodium salt dihydrate (Na_2_EDTA) and 10 mM ammonium acetate in milli-Q water) at pH 3 with a flow rate of 0.8 mL/min was used with a sample injection volume of 50 µL. Quantification was performed using different concentrations of ascorbic acid standard dissolved in the extraction buffer and injected into the HLPC system to generate a calibration curve.

### 2.5. Phytochemical Compounds’ Analysis

#### 2.5.1. Carotenoid Analysis

In this study, carotenoids were extracted and quantified as described by Vancoillie et al. [24]. Briefly, the entire extraction procedure was performed under red light because of the light sensitivity of the carotenoids. The ball-milled sample (100 mg) was weighed into a dark Eppendorf tube, and 1 mL of extraction solvent (99% ethanol containing 0.1% *w*/*v* butylhydroxytoluene) was added, followed by a known concentration of echinenone (dissolved in extraction solvent) as the internal standard. These components were mixed by vortexing and placed on an end-over-end rotor for 15 min at 4 °C, after which it was centrifuged at 12,300× *g* and 4 °C for 5 min (Microfuge 22R, Beckman Coulter Inc., Indianapolis, IN, USA) and the supernatant was collected. The pellet obtained after centrifugation was further extracted twice using the same procedure, and the supernatants from the three extractions from each sample were pooled. The extract (4.5 mL) was poured into an amber glass jar containing Ambersep 900-OH resin and stirred for 30 min at 4 °C to remove chlorophyll, which would have interfered with the carotenoid peaks. The resultant solution was filtered through a Chroma-fil PET- 20/25 filter into amber HPLC vials for analysis.

The quantification of the carotenoids was performed with a YMC C30 column (150 × 4.6 mm, 3 μm particle size, YMC CO., Ltd., Kyoto, Japan) on an HPLC with a diode array detector (1200 Series, Agilent Technologies, Diegem, Belgium). Separation of the carotenoids was effected by an elution programme at a flow rate of 1 mL/min with three solvents, methanol, MTBE (methyl-t-butyl ether), and Milli-Q water with the following steps: 0–10 min: 95% methanol, 0–5% milli-Q water and 0–3% MTBE; 10–20 min: 95–78.3% methanol, 3–21% MTBE and 2–0.7% milli Q water; 20–23 min: 78.3–50% methanol, 21–50% MTBE; 23–27 min: 50% methanol and 50% MTBE; and 27–32 min: 95% methanol and 5% milli-Q water. The column temperature was set at 25 °C, and the detector was set at the wavelengths of maximal absorbance for the carotenoids in ethanol as reported by Britton et al. [25], i.e., 440 nm for violaxanthin and neoxanthin, 445 nm for lutein, 450 nm for β-carotene and 461 nm for echinenone. Standard curves of violaxanthin, neoxanthin, lutein, and β-carotene were used to quantify the specific carotenoids.

#### 2.5.2. Total Polyphenol and Total Flavonoid Analysis

Prior to the analysis of total polyphenol content (TPC) and total flavonoid content (TFC), extraction of the phenolics was performed as described by Agcam et al. [26] with a few modifications. Briefly, we accurately weighed 100 mg of the sample into centrifuge tubes and placed these in an ice bath. To the latter, 15 mL of acidified methanol (80% methanol; 0.4% HCL; 19.6% milli-Q water) was added, vortexed for 30 s, and placed on an end-over-end rotor for 45 min. This mixture was centrifuged at 3020× *g* (Avanti JXN-26, Beckman Coulter Inc, Indianapolis, IN, USA) for 15 min at 4 °C, and the supernatant was filtered through a chroma-fil filter (0.45 µm) into a glass tube.

The total phenolic content (TPC) analysis was performed as described by Agcam et al. [26] with a few modifications. From the supernatant or standard solution, 100 µL was pipetted into a reaction tube followed by the addition of 500 µL of Folin–Ciocalteu reagent and vortexed for five seconds. It was then left to stand for five minutes before adding 1 mL of saturated sodium carbonate solution (20% *w*/*v*) and 8.4 mL of milli-Q water. The mixture was left to react for 1.5 h at 25 °C in the dark, and its absorbance was then measured at 765 nm using an Ultrospec 2100 pro UV-Vis spectrophotometer (GE Healthcare, Chicago, IL, USA). The TPC was calculated and expressed in gallic acid equivalents (GAE) per gram dry weight with gallic acid as the standard ($y[Abs]=0.0012x+0.0099;\;R^{2}=0.999$).

The TFC was quantified using a method elaborated by Hossain et al. [27] with slight modifications. Briefly, 500 µL of the extract or standard solution was pipetted into a reaction tube followed by 200 µL of aluminium chloride (10% *w*/*v*). The mixture was vortexed and 200 µL of potassium acetate (1 M) was added before dilution with 5 mL of milli-Q water. The resultant solution was left to stand for 30 min at 25 °C, and its absorbance was read at 415 nm using an Ultrospec 2100 pro-UV-Vis spectrophotometer (GE Healthcare, Chicago, USA). The flavonoid content was computed based on a quercetin standard, and the results were expressed in Quercetin Equivalents (QEs) per gram dry weight ($y[Abs]=0.0056x+0.0077;\;R^{2}=0.999$).

#### 2.5.3. Chlorophyll Analysis

Chlorophylls were extracted with ethanol (>95%) from a ball-milled sample under red light. To 40 mg of the sample, 10 mL of ethanol was added, vortexed for 30 s, and placed on an end-over-end rotor for 30 min at 4 °C. The resultant mixture was centrifuged using a Sigma 4-16KS centrifuge (Osterode am Harz, Germany) for 10 min at 12,300× *g* and 4 °C, and the supernatant was filtered into amber HPLC vials through a 0.25 µm membrane. Analysis was performed using a YMC C30 column (150 × 4.6 mm, 3 μm particle size, YMC CO., Ltd., Kyoto, Japan) connected to an HPLC with a diode array detector (1200 Series, Agilent Technologies, Diegem, Belgium). The same gradient elution programme used for carotenoid analysis was applied to separate the chlorophylls (Section 2.5.1). The diode array detector was set at wavelengths for the maximum absorption of chlorophylls in ethanol, which are 664 nm and 649 nm for chlorophyll a and b, respectively [28]. Using standard curves of the two chlorophylls (a and b), the amounts present in the samples were calculated and expressed in milligrams per 100 g dry weight.

### 2.6. Statistical Data Analysis

For each of the nine leafy vegetables, three representative samples were analysed independently for all compounds. The means of the representative samples were statistically compared using one-way ANOVA (analysis of variance) and Tukey’s tests with JMP Pro. 17 (SAS, Institute. Inc, Cary, NC, USA) software at a significance level of α = 0.05.

## 3. Results and Discussion

### 3.1. Macronutrient Composition of the Leafy Vegetables

The macronutrient composition (moisture, protein, dietary fibre, ash and starch) of the selected African leafy vegetables is shown in Table 1, and the obtained quantities significantly differed among the vegetables (*p* < 0.05). The leafy vegetables generally had a high moisture content (>80 g/100 g DW), explaining their high perishability. The amount of crude protein varied from 14.96 ± 0.05 (Malakwang) to 39.53 ± 0.07 g/100 g DW (nightshade). Nightshade, African nightshade, and pumpkin leaves had the highest amounts of true protein, i.e., 33.41 ± 0.40, 31.29 ± 0.39, and 30.36 ± 0.12 g/100 g DW, respectively. Reminiscent of the crude protein, Malakwang had a significantly (*p* < 0.05) lower true protein content (13.27 ± 0.05 g/100 g DW). The observed ranges of crude protein and moisture were comparable to the ranges reported by Ntuli [4], Gupta et al. [29] and Van Jaarsveld et al. [5]. However, Gupta et al. [29] reported crude protein values that were about 19% and 25% lower in pumpkin leaves and *Amaranthus* sp., respectively, compared to those in this study. Anju et al. [7] found a lower crude protein content range (4.55–22.32 g/100 g DW) in traditional leafy vegetables, although Malakwang’s protein content (13.27 g/100 g DW) was within the reported range.

The difference between the crude protein and true protein reflects the presence of non-protein nitrogen ranging from 1.67 (cowpea leaves) to 8.24 g/100 g DW (Spider plant). To our knowledge, this is the first study to quantify the true protein content in African leafy vegetables, which is highly relevant since the non-protein nitrogen is part of the crude protein, resulting in overestimation of the protein content.

Substantial amounts of dietary fibre were noticed in the leafy vegetables, with the highest found in Malakwang (43.39 ± 0.25 g/100 g DW) and the lowest in nightshade (28.67 ± 0.13 g/100 g DW). The total dietary fibre for cowpea leaves and *Amaranthus* sp. was comparable to the findings of Van Jaarsveld et al. [5]. However, Van Jaarsveld et al. [5] reported about 17%, 31% and 24% lower dietary fibre for black nightshade, pumpkin leaves, and spider plant, respectively, compared to the present study. These differences are somewhat expected because dietary fibre depends not only on the plant species but also on the maturity level of the plant [30], and the vegetables are typically harvested at different maturity stages.

The total starch content was generally low (<6 g/100 g DW) in the vegetables, while the ash content ranged from 7.68 ± 0.13 g/100 g DW (Malakwang) to 20.05 ± 0.16 g/100 g DW (red amaranthus). In other studies [4,5,7], the total carbohydrate content in the leaves was determined instead of that of only starch; hence, no comparisons could be made. Nonetheless, the low amounts of starch are somewhat expected since leaves are known to be photosynthetic sites in plants and not food storage sites.

Pulses such as common beans and soybeans are important sources of protein to the majority of the Sub-Saharan African populations and are known to be high-protein plant-based foods with a 16–25% dry weight [31] and 34–43% [32] of protein, respectively. Compared to these pulses, the analysed leafy vegetables have crude protein contents comparable to that of soybeans and higher than the amounts in common beans on a dry weight basis. Therefore, the dried leafy vegetable products can be considered good alternative protein sources, although the protein quality (amino acid composition) and digestibility are yet to be determined.

In addition, commonly consumed cereals such as wheat, sweet corn and whole-meal rice reportedly contain about 15.8, 5.6, 9.2 and 9.2% dietary fibre (dry weight), respectively [33], while pulses including chickpeas, dry peas, and beans contain about 24% dietary fibre on average [34]. This confirms that leafy vegetables are excellent sources of dietary fibre compared to commonly consumed cereals and pulses. The consumption of such dietary fibre-rich foods is associated with health benefits including improved gut motility, insulin sensitivity, colonic health, and gut microflora, among others [35], making leafy vegetables a vital component of a healthy diet. In addition to the high dietary fibre, starch was found in low concentrations, which is beneficial as most common staple foods in Africa are rich in starch.

### 3.2. Micronutrient Composition of the Leafy Vegetables

#### 3.2.1. Mineral Profile and Content

The mineral composition of the analysed vegetables (macro and microelements) expressed in mg/100 g DW is shown in Table 2. Among the macroelements, K and Ca had the highest concentration in all the vegetables except for pumpkin leaves. The K content ranged from 609.44 ± 29.07 mg/100 g DW (Malakwang) to 3183.60 ± 329.95 mg/100 g DW (red amaranthus), while that of Ca ranged from 629.50 ± 3.49 mg/100 g DW (pumpkin leaves) to 3395.46 ± 59.21 mg/100 g DW (red amaranthus). Overall, K and Ca were followed by Mg, the content of which showed significant differences, between 323.74 ± 11.98 mg/100 g DW for Malakwang and 1428.10 ± 27.96 mg/100 g DW for red amaranthus. The phosphorus content also differed among the vegetables, ranging from 258.41 ± 9.13 mg/100 g DW in Malakwang to 1104.17 ± 6.72 mg/100 g DW in pumpkin leaves.

This study quantified two microelements of nutritional relevance (Fe and Zn). Fe had comparatively higher concentrations than Zn in all the vegetables, at between 14.05 ± 0.26 mg/100 g DW in collard leaves and 78.30 ± 1.16 mg/100 g DW in green amaranthus. The concentrations of Zn were generally low and differed slightly among the vegetables, within a range of 2.27 ± 1.33 to 5.94 ± 0.93 mg/100 g DW. The low concentrations of Zn are consistent with those reported in other studies conducted on leafy vegetables [5,36,37,38,39].

The mineral contents for cowpeas leaves (Mg, Ca, P, and Zn) and spider plant (Mg and Zn) were comparable with the amounts reported by Thovhogi et al. [36]. The amounts of Zn, Fe and Mg contents were comparable with the findings of Schönfeldt and Pretorius [37] for *Amaranthus* sp. In contrast, the amount of Ca, Mg, P, and Fe observed in pumpkin leaves deviated from those reported by Thovhogi et al. [36]. Schönfeldt and Pretorius [37] reported about 4 times higher mineral contents in pumpkin leaves (Ca, Mg and Fe) and about 1.5–2.5 times higher content in cowpea leaves (Fe and Mg) and spider plant (Fe and Ca) compared to the present study. Jiménez-Aguilar and Grusak [39] analysed spider plant accessions of different origins and reported amounts of Ca that were about 50% lower than the quantities for spider plant in this study. These disagreements are probably due to differences in the growing areas and agricultural practises, such as using manure/fertilisers in the growing fields. Malakwang (*Hibiscus sabdariffa*), the mineral profile of which has not been previously studied, had the lowest concentration of both macro- and microelements, which has also been reflected in its total ash content, making this vegetable a poor source of minerals.

Ca and Mg are essential micro-components of bones and teeth. Considering a recommended average consumption of about 240 g/day of vegetables on a fresh-weight basis [40], red amaranthus, green amaranthus, and collard leaves can potentially contribute to approximately 90% of the population reference intake (PRI) of calcium (1000 mg/day) for adults [41]. Spider plant, Malakwang, and cowpea leaves have the potential to contribute to more than 60% (Table S1). On the other hand, with an estimated adequate intake (AI) of 300 mg/day for Mg in an adult female [42], the two amaranthus species can contribute to more than 100% of the required Mg. Except for Malakwang and African nightshade, all the other vegetables have the potential to provide between 45 and 82% of the AI when consumed in similar proportions (240 g). Therefore, the analysed vegetables can be valuable sources of Ca and Mg. However, some antinutrients in leafy vegetables, such as oxalates, form insoluble salts with minerals and reduce their bioaccessibility [43]. Therefore, attempts should be made to reduce these compounds and improve bioaccessibility.

The ratio of dietary calcium to phosphorus (Ca:P) is suggested to be an essential factor for bone growth and maintenance. A low Ca:P ratio (<0.5) may result in the release of calcium from the bones through bone resorption to balance the calcium levels in the blood [44,45], which affects bone density. The Ca:P ratios in the studied ALVs were higher than 2 (2.2–8.3), except for pumpkin leaves, which had a ratio of 0.57. Hence, consumption of these vegetables would promote proper bone formation and maintenance.

According to the EFSA Panel on Dietetic Products, Nutrition and Allergies [46], the PRI of Zn (reference weight 58.5 kg) is about 10.1 mg/day. On that note, the analysed vegetables can contribute less than 21% of the PRI, suggesting that they are not good sources of Zn. Iron is a crucial element in oxygen transportation and a deficiency of it, anaemia, is common in poor households with limited access to animal-based foods (rich in heme iron). Considering an adequate intake (AI) of 58.8 mg of Fe/day for an adult female [47] for foods with 5% iron bioavailability, 240 g of green amaranthus, cowpeas, and spider plant can potentially contribute 48.8, 40.6, and 37.3% (Table S1) of the AI, respectively. Therefore, the vegetables mentioned above can be fairly good sources of iron. As it is known that plants may contain components that chelate minerals, it would be worthwhile to investigate the mineral bioaccessibility in the selected leafy vegetables to better understand the leafy vegetable mineral contributions.

#### 3.2.2. Vitamin C Content

The total vitamin C content significantly varied among the analysed vegetables (*p* < 0.05), as shown in Table 3. The highest concentration was found in Malakwang (253.2 ± 13.0 mg/100 g DW), followed by collard leaves (213.3 ± 4.0 mg/100 g DW), and the lowest was observed in spider plant (23.8 ± 1.3 mg/100 g DW). Three vegetables, spider plant, African nightshade, and green amaranthus, had quite low quantities of vitamin C (<37 mg/100 g DW), whereas the other vegetables had quantities above 115 mg/100 g DW.

The cowpea leaves’ vitamin C content was comparable to the amounts reported by Wawire et al. [48]. However, Van Jaarsveld et al. [5] reported about 2.8 times lower vitamin C in *Amaranthus* sp., nightshade, cowpeas, spider flower, and pumpkin leaves. On the other hand, the amount of vitamin C in this study (green amaranthus) was about 7 and 10 times lower than the amounts reported by Medoua and Oldewage-Theron [49] and Ejoh et al. [50], respectively, in *Amaranthus* sp. In the present study, the samples were collected from the market shelves, which could have caused some vitamin C degradation before sample collection, explaining substantial differences from the literature values.

For an estimated recommended nutrient intake (RNI) of about 120 mg of vitamin C for an average adult (≈70 kg) [51], Malakwang and collard leaves can contribute about 70% of the RNI for 240 g (fresh weight) of consumption, whereas pumpkin leaves can contribute about 48% (Table S1). On that note, the above-mentioned vegetables can be considered fairly good sources of vitamin C, provided the appropriate processing and storage conditions are emphasised to limit vitamin C degradation.

### 3.3. Phytochemical Composition of the Leafy Vegetables

#### 3.3.1. Carotenoid Profile and Content

**Figure 1:**
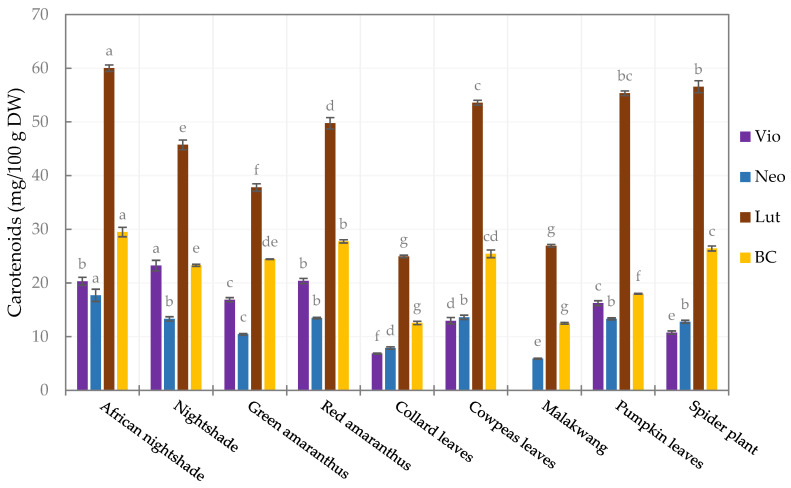
Carotenoid profile of the leafy vegetables (Vio, violaxanthin; Neo, neoxanthin; Lut, lutein; BC, β-carotene). Bars in the same legend with different letters are significantly different (*p* < 0.05) (*n* = 3).

The carotenoid profile (Figure S1) of the leafy vegetables comprised lutein (42.2–58.6%), β-carotene (17.5–27.3%), neoxanthin (11.6–15.1%), and violaxanthin (0.0–22.0%), and their concentrations varied (*p* < 0.05) among the vegetables (Figure 1). Among the quantified carotenoids, lutein had the highest concentration in all the vegetables, ranging from 24.9 ± 0.24 (collard leaves) to 60.0 ± 0.6 mg/100 g DW (African nightshade). The second highest was β-carotene, with the highest quantities observed in African nightshade and red amaranthus (29.5 ± 0.89 and 27.8 ± 0.31 mg/100 g DW, respectively) and the lowest contents in Malakwang and Collard leaves (12.5 ± 0.2 and 12.6 ± 0.3 mg/100 g DW, respectively). Similar trends were reported by Raju et al. [52] and Song et al. [53] in green leafy vegetables and Bunea et al. [54] in spinach.

African nightshade had the highest contents of violaxanthin and neoxanthin, at 20.3 ± 0.7 and 17.7 ± 1.1 mg/100 g DW, respectively. Notably, the violaxanthin content was below quantifiable levels in Malakwang, which also had the lowest quantity of neoxanthin (5.9 ± 0.1 mg/100 g DW). A similar observation was made in *Hibiscus cannabinnus* [52], also belonging to the *Malvaceae* family. Malakwang and collard leaves had the lowest concentration of all the quantified carotenoids, and consequently, the least total carotenoid content (the sum of all the quantified carotenoids), which was 46.0 ± 0.4 and 52.2 ± 0.7 mg/100 g DW, respectively (Table 3). Therefore, it can be concluded that these two vegetables are not good sources of these health-beneficial compounds. Significant differences in the carotenoid content (individual and total carotenoids) among vegetables of the same family (*Amaranthaceae* and *Solanaceae*) were noticed, suggesting that the carotenoid content is species-dependent.

Comparable results were obtained for violaxanthin in pumpkin leaves and nightshade by Raju et al. [52], as well as for lutein, violaxanthin, and neoxanthin in green amaranthus and neoxanthin, lutein and β-carotene in cowpea leaves by Wen Lee et al. [55]. The concentration of β-carotene in green amaranthus was about 35% lower than the amount reported by Wen Lee et al. [55]. In the pumpkin leaves, the β-carotene was about 71% lower than what Chandrika et al. [56] reported. Furthermore, the β-carotene and lutein in nightshade was about half of the amount reported by Raju et al. [52], while the amounts of the same carotenoids in pumpkin leaves in our study were about 50% higher. For other leafy vegetables, moringa and cassava leaves were reported to have about three times more carotenoids [55] than the African nightshade, which had the highest content for all the carotenoids in our study. This study is the first to profile carotenoids in Malakwang and African nightshade.

Among the quantified carotenoids, β-carotene is known to have provitamin A activity and can therefore be converted to vitamin A within the body. Vitamin A is an essential micronutrient for maintaining sight, immunity, and reproduction, with a population reference intake (PRI) of about 700 µg RE (retinol equivalents) for adults [57]. Considering an estimated consumption of 240 g of fresh leaves, the vegetables have the potential to contribute more than 100% of the PRI, except for Malakwang, which falls slightly below 100% of the PRI (Table S1). In addition, these vegetables contain substantial amounts of lutein, a macular pigment in the eyes [58], and their consumption could be vital for protection against ocular diseases and age-related macular degeneration. However, it should be noted that the low-fat content and encapsulation of carotenoids in the chloroplast might limit their bioaccessibility and bioavailability. To improve bioaccessibility, some oil can be added during or after appropriate processing before consumption to promote the release of carotenoids [59]. This would make the vegetables reliable sources of pro-vitamin A in the form of β-carotene and the macular pigment lutein.

#### 3.3.2. Total Polyphenol Content and Total Flavonoid Content

The total polyphenol content (TPC) in the different leafy vegetable extracts was between 16.8 ± 1.3 (pumpkin leaves) and 42.5 ± 0.3 mg GAE/g DW (Malakwang), as shown in Table 3. Relatively small differences in the total flavonoid content (TFC) were observed, with the highest content in Malakwang (16.3 ± 0.5 mg QE/g DW) and the lowest in collard leaves (11.5 ± 0.2 mg QE/100 g DW). The TPC and TFC were in the range of contents found for different *Amaranthus* sp. by Sarker and Oba [9]. The observed ranges were comparable with the findings of Nyero et al. [60] for wild leafy vegetables and Jiménez-Aguilar and Grusak [39] for spider plant. Polyphenols, like other phytochemicals, vary widely in plants due to differences in plant species, growing conditions, maturity, and post-harvest handling [13], which could somewhat explain the observed variations among the vegetables including those from the same family.

#### 3.3.3. Chlorophyll Content

**Figure 2:**
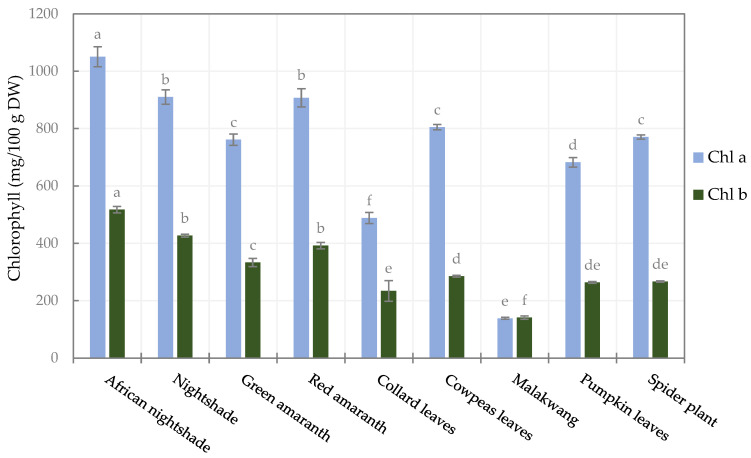
Chlorophyll content of the selected leafy vegetables. (Chl a and Chl b are chlorophyll a and b, respectively). Bars of the same legend with different letters are significantly different (*p* < 0.05) (*n* = 3).

Apart from red amaranthus, the analysed AILVs were green; chlorophyll is the pigment responsible for this greenness. Two major chlorophylls were quantified, i.e., chlorophyll a and b, and they varied significantly (*p* < 0.05) among the vegetables, as shown in Figure 2. The highest quantities of chlorophyll a and b were noticed in African nightshade, which were 1050.6 ± 34.8 and 517.4 ± 10.9 mg/100 g DW, respectively. The lowest contents were found in Malakwang, which were 138.5 ± 3.4 and 140.9 ± 5.8 mg/100 g DW for chlorophyll a and b, respectively.

After the two *Solanaceae* species, red amaranthus had the highest total chlorophyll content (1299.3 mg/100 g DW) despite being red, implying that the chlorophyll in red amaranthus is visually masked by other pigments such as anthocyanins and betalains [61]. To our knowledge, chlorophyll has not been quantified or profiled in the vegetable species studied in this work. Compared to other leafy vegetables, the concentrations of chlorophylls are close to those reported by Nartnampong et al. [62] in Thai green leafy vegetables. However, in the current study, the amounts of the chlorophylls (a and b) observed are about twice as high as those reported by Sarker and Oba, [9] in red morph amaranthus leafy vegetables and by Sarker et al. [61] in *Amaranthus tricolor* and *Amaranthus lividus*.

Generally, chlorophyll a was the predominant form, and the ratio of chlorophyll a to b varied between 2.1 (Collard leaves, African nightshade, and nightshade) and 2.8 (spider plant and cowpeas leaves), except for Malakwang, the ratio of which was as low as 1. This agrees with the ratios reported by Sarker and Oba [9] and Sarker et al. [61] in *Amaranthus* sp.

Chlorophyll derivatives have a bioactivity linked to their ability to act as antioxidants and antimutagens by scavenging free radicals and preventing DNA damage [63]. Some of the chlorophyll derivatives have been shown to regulate the gut microbiota and prevent intestinal inflammation in mice [64]. In addition to the other phytochemicals discussed, the chlorophyll in these vegetables can potentially impart extra health benefits.

## 4. Conclusions

Some of the health-related challenges in Uganda include protein and micronutrient-based malnutrition (poor populations) and increased incidences of non-communicable diseases (NCDs), especially in the relatively rich populations (middle class). With an aim to provide science-based and sustainable solutions, this study documents the nutrient and phytochemical composition of nine African leafy vegetables commonly consumed in Uganda. The selected leafy vegetables are highlighted as potential contributors to the much-needed dietary fibre and protein as an alternative to the expensive animal-based protein in the diets. African nightshade, *Amaranthus* sp., cowpeas leaves, and spider plant contain substantial quantities of β-carotene (provitamin A), calcium (Ca) and iron (Fe), hence being essential in combating the related micronutrient deficiencies, which are prevalent in sub-Saharan Africa. Malakwang and collard leaves are relatively good sources of vitamin C, provided the appropriate post-harvest handling practises are applied to minimise possible losses.

The selected African leafy vegetables are also viable sources of natural antioxidant compounds (phytochemicals) such as carotenoids and polyphenols, which have been associated with reduced risk of NCDs, making them health-beneficial foods. Despite Malakwang having a relatively low content of minerals, carotenoids and protein, it contains considerable quantities of polyphenols and dietary fibre, making it a health-promoting vegetable.

The African leafy vegetables are usually thermally processed (cooked) to improve their palatability and dried to improve shelf stability. This processing might influence not only the concentration of the nutrients and phytochemicals but also their bioaccessibility. Therefore, evaluating the impact of processing (cooking and drying) on the nutrients and phytochemicals would be interesting as a follow-up to the present study.

## Figures and Tables

**Table 1:** Macronutrient composition of the selected African leafy vegetables.

Vegetable	Moisture (g/100 g FW)	Dietary Fibre	Crude Protein	True Protein	Total Ash	Total Starch
		g/100 g DW				
African nightshade	89.51 ± 0.60 ^a^	35.78 ± 0.18 ^bc^	37.53 ± 0.21 ^b^	31.29 ± 0.39 ^b^	17.18 ± 0.45 ^bc^	0.47 ± 0.05 ^f^
Nightshade	88.82 ± 0.18 ^a^	28.67 ± 0.13 ^e^	39.53 ± 0.07 ^a^	33.41 ± 0.40 ^a^	13.91 ± 0.29 ^d^	1.74 ± 0.07 ^c^
Green amaranthus	84.72 ± 0.51 ^cd^	36.91 ± 0.12 ^b^	30.63 ± 0.44 ^e^	27.76 ± 0.38 ^de^	17.62 ± 0.08 ^b^	1.64 ± 0.10 ^c^
Red amaranthus	88.79 ± 0.26 ^a^	36.27 ± 0.35 ^bc^	32.38 ± 0.72 ^d^	26.77 ± 0.14 ^e^	20.04 ± 0.16 ^a^	0.77 ± 0.04 ^e^
Collard leaves	83.81 ± 0.35 ^d^	35.02 ± 0.41 ^c^	26.38 ± 0.18 ^f^	21.17 ± 0.26 ^f^	13.03 ± 0.14 ^e^	1.67 ± 0.12 ^c^
Cowpeas leaves	85.55 ± 0.49 ^bc^	30.45 ± 0.29 ^de^	31.06 ± 0.45 ^e^	29.38 ± 0.26 ^c^	10.69 ± 0.07 ^g^	5.11 ± 0.13 ^a^
Malakwang	86.29 ± 0.48 ^b^	43.39 ± 0.25 ^a^	14.96 ± 0.05 ^g^	13.27 ± 0.05 ^g^	7.68 ± 0.13 ^h^	2.11 ± 0.07 ^b^
Pumpkin leaves	85.58 ± 0.33 ^bc^	30.09 ± 0.19 ^e^	33.84 ± 0.42 ^c^	30.36 ± 0.12 ^bc^	11.33 ± 0.31 ^f^	0.96 ± 0.03 ^d^
Spider plant	85.36 ± 0.53 ^bc^	32.41 ± 0.20 ^d^	36.43 ± 0.16 ^b^	28.19 ± 0.31 ^d^	16.74 ± 0.16 ^c^	0.96 ± 0.03 ^d^

Values are the mean of three replicates ±the standard deviations. The values followed by a different letter(s) within a column significantly differ from each other (*p* < 0.05) (*n* = 3). FW—fresh weight; DW—dry weight.

**Table 2:** Mineral content (macro and microelements) of the selected African leafy vegetables expressed in mg/100 g dry weight.

Leafy Vegetable	K	Ca	Mg	P	Fe	Zn
African nightshade	1562.74 ± 374.29 ^c^	1265.35 ± 67.67 ^e^	423.83 ± 42.58 ^ef^	548.34 ± 5.39 ^e^	64.85 ± 4.17 ^bc^	3.12 ± 0.53 ^ef^
Nightshade	2458.51 ± 333.58 ^ab^	1689.89 ± 16.14 ^d^	513.83 ± 1.30 ^d^	542.39 ± 2.23 ^e^	29.03 ± 0.98 ^e^	4.03 ± 0.06 ^cd^
Green amaranthus	2854.68 ± 337.63 ^ab^	2466.37 ± 129.83 ^b^	1322.95 ± 11.72 ^b^	566.23 ± 1.97 ^e^	78.30 ± 1.16 ^a^	5.23 ± 0.02 ^ab^
Red amaranthus	3183.60 ± 329.95 ^a^	3395.46 ± 59.21 ^a^	1428.10 ± 27.96 ^a^	737.97 ± 16.65 ^c^	42.32 ± 1.36 ^d^	5.94 ± 0.49 ^a^
Collard leaves	2074.52 ± 314.03 ^bc^	2361.28 ± 18.51 ^b^	467.89 ± 2.34 ^de^	634.05 ± 4.94 ^d^	14.05 ± 0.26 ^g^	2.74 ± 0.07 ^fg^
Cowpeas	1533.44 ± 83.99 ^c^	1795.44 ± 51.62 ^d^	397.91 ± 6.77 ^f^	494.26 ± 14.79 ^f^	68.77 ± 2.39 ^b^	3.53 ± 0.17 ^de^
Malakwang	609.44 ± 29.07 ^d^	2151.95 ± 75.33 ^c^	323.74 ± 11.98 ^g^	258.41 ± 9.13 ^g^	16.99 ± 0.80 ^g^	2.27 ± 0.12 ^g^
Pumpkin leaves	2447.39 ± 59.56 ^b^	629.50 ± 3.49 ^f^	434.18 ± 3.17 ^ef^	1104.17 ± 6.72 ^a^	22.38 ± 1.51 ^f^	5.83 ± 0.02 ^a^
Spider plant	2402.75 ± 157.13 ^b^	2004.62 ± 9.67 ^c^	700.76 ± 2.62 ^c^	923.33 ± 1.62 ^b^	62.42 ± 0.50 ^c^	4.57 ± 0.05 ^bc^

Values are the mean of three replicates ± the standard deviations. The values followed by a different letter or different letters within a column significantly differ from each other (*p* < 0.05) (*n* = 3).

**Table 3:** Total polyphenol, total flavonoids, vitamin C, β-carotene, total carotenoids, and total chlorophyll in the selected African leafy vegetables.

Leafy Vegetable	TPC (mg GAE/g DW)	TFC (mg QE/g DW)	Vitamin C (mg/100 g DW)	β-Carotene (mg/100 g DW)	Total Carotenoids (mg/100 g DW)	Total Chlorophyll (mg/100 g DW)
African nightshade	35.7 ± 0.4 ^b^	14.7 ± 0.7 ^b^	28.2 ± 0.5 ^f^	29.5 ±0 0.9 ^a^	127.5 ± 1.7 ^a^	1568.0 ± 42.0 ^a^
Nightshade	24.1 ± 0.7 ^d^	14.8 ± 0.3 ^b^	134.0 ± 8.9 ^d^	23.3 ± 0.2 ^e^	105.6 ± 1.9 ^c^	1337.0 ± 29.4 ^b^
Green amaranth	21.6 ± 0.8 ^ef^	13.1 ± 0.2 ^c^	36.0 ± 2.2 ^f^	24.4 ± 0.1 ^de^	89.6 ± 1.3 ^d^	1094.4 ± 32.4 ^c^
Red amaranth	27.1 ± 0.3 ^c^	12.3 ± 0.1 ^d^	125.8 ± 4.7 ^de^	27.8 ± 0.3 ^b^	111.4 ± 1.6 ^b^	1299.3 ± 40.3 ^b^
Collard leaves	20.8 ± 0.6 ^f^	11.5 ± 0.2 ^e^	213.3 ± 4.0 ^b^	12.6 ± 0.3 ^g^	52.2 ± 0.7 ^e^	722.6 ± 20.9 ^e^
Cowpeas leaves	20.6 ± 0.8 ^f^	15.2 ± 0.2 ^b^	115.8 ± 2.3 ^e^	25.4 ± 0.7 ^cd^	105.6 ± 0.8 ^c^	1090.5 ± 10.7 ^c^
Malakwang	42.5 ± 0.3 ^a^	16.3 ± 0.5 ^a^	253.2 ± 13.0 ^a^	12.5 ± 0.2 ^g^	46.0 ± 0.4 ^f^	279.3 ± 3.0 ^f^
Pumpkin leaves	16.8 ± 1.3 ^g^	12.2 ± 0.4 ^de^	166.7 ± 4.0 ^c^	18.0 ± 0.1 ^f^	102.9 ± 1.0 ^c^	946.1 ± 17.7 ^d^
Spider plant	23.7 ± 1.0 ^de^	16.1 ± 0.9 ^a^	23.8 ± 1.3 ^f^	26.4 ± 0.5 ^c^	106.5 ± 1.8 ^c^	1037.6 ± 5.4 ^c^

Values are the mean of three replicates ± the standard deviations. TPC, total polyphenol content; TFC, total flavonoid content. The values followed by a different letter or different letters within a column significantly differ from each other (*p* < 0.05) (*n* = 3).

## Data Availability

The raw data supporting the conclusions of this article will be made available by the authors on request.

## Supplementary Materials

The following supporting information can be downloaded at https://www.mdpi.com/article/10.3390/foods14081304/s1.

## Abbreviations

The following abbreviations are used in this manuscript:

ALVs	African leafy vegetables
BC	β-carotene
Chl	Chlorophyll
DW	Dry weight
FW	Fresh weight
GAE	Gallic acid equivalent
Lut	Lutein
NCDs	Non-communicable diseases
Neo	Neoxanthin
QE	Quercetin equivalent
TFC	Total flavonoid content
TPC	Total polyphenol content
Vio	Violaxanthin
