# Supplementary material for: Nutrient and Phytochemical Composition of Nine African Leafy Vegetables: A Comparative Study
Source: Foods. 2025 Apr 9;14(8):1304. doi: 10.3390/foods14081304 (PMC12027459; doi:10.3390/foods14081304)
Supplement: Supplementary file 1 [file foods-14-01304-s001.zip › foods-3508436-supplementary.pdf]

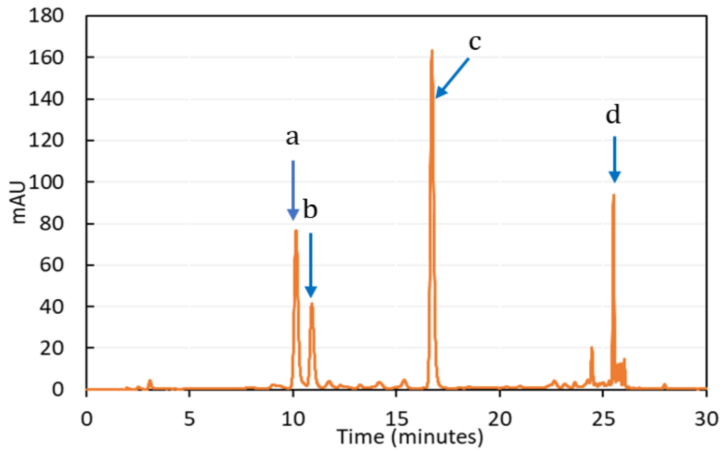

Figure S1: Exemplary chromatogram for the carotenoid profile for green amaranthus with identification of the major peaks: a-violaxanthin, b-neoxanthin, c-lutein, β-carotene

Table S1: Reference intake percentage contribution of the micronutrients for a 240 g (fresh weight) portion of the African leafy vegetables

**Leafy vegetable**	**Vitamin C**	**β-carotene**	**Ca**	**Mg**	**Fe**	**Zn**
African nightshade	5.9	176.7	31.8	35.6	27.8	8.0
Nightshade	29.9	148.7	45.3	45.9	13.2	11.0
Green amaranthus	11.0	213.4	90.5	161.8	48.8	19.6
Red amaranthus	28.2	177.7	91.3	128.0	19.4	16.3
Collard leaves	69.1	116.2	91.8	60.6	9.3	10.9
Cowpeas leaves	33.5	210.1	62.3	46.0	40.6	12.5
Malakwang	69.4	97.9	70.8	35.5	9.5	7.6
Pumpkin leaves	48.1	148.3	21.8	50.1	13.2	20.6
Spider plant	7.0	221.2	70.5	82.1	37.3	16.4
PRI/AI/RNI (mg/day)	120	4200	1000	300	58.8	10.1

PRI is population reference intake; AI is adequate intake; RNI is recommended nutrient intake.
